# Successful Control of Chemotherapy-Induced Breakthrough Hemolysis With Ravulizumab in a Patient With Paroxysmal Nocturnal Hemoglobinuria During Carboplatin-Pemetrexed Treatment for Lung Adenocarcinoma

**DOI:** 10.7759/cureus.88219

**Published:** 2025-07-18

**Authors:** Atsushi Takahata, Keisuke Tanaka, Shigeo Toyota

**Affiliations:** 1 Department of Hematology, Yokosuka Kyosai Hospital, Yokosuka, JPN; 2 Department of Hematology, Institute of Science Tokyo, Tokyo, JPN

**Keywords:** chemotherapy-induced hemolysis, complement c5 inhibition, lung adenocarcinoma, paroxysmal nocturnal hemoglobinuria, ravulizumab

## Abstract

Paroxysmal nocturnal hemoglobinuria (PNH) is a rare hematologic disorder characterized by complement‑mediated intravascular hemolysis. Although breakthrough hemolysis (BTH) is typically triggered by infections or surgery, chemotherapy-induced BTH is seldom reported, and optimal management strategies during cytotoxic cancer therapy remain undefined. We report a 52‑year‑old man with longstanding PNH who developed stage IVA epidermal growth factor receptor‑mutated lung adenocarcinoma. After discontinuing first‑line osimertinib due to diarrhea, second‑line carboplatin-pemetrexed (chemotherapy regimen consisting of carboplatin and pemetrexed) induced severe BTH, evidenced by lactate dehydrogenase rising to 2,462 U/L and hemoglobin (Hb) dropping to 4.6 g/dL. Introduction of ravulizumab promptly normalized lactate dehydrogenase (<250 U/L), raised Hb to 10.5 g/dL, and suppressed total hemolytic complement activity (<14 U/mL). Although mild hemolysis recurred before subsequent cycles, administering ravulizumab before each chemotherapy session prevented further episodes. The patient completed 14 cycles without transfusion or thrombosis and achieved a progression-free survival (PFS) of two years and five months, far beyond the ~5.5 month median for this regimen. Personalized scheduling of ravulizumab enabled uninterrupted cytotoxic chemotherapy by effectively managing BTH, suggesting that sustained complement C5 inhibition may confer oncologic benefits. Prospective studies are warranted to evaluate the broader impact of complement blockade in patients with PNH and malignancy.

## Introduction

Paroxysmal nocturnal hemoglobinuria (PNH) is a clonal hematopoietic disorder caused by a somatic phosphatidylinositol glycan class A mutation, leading to a deficiency of glycosylphosphatidylinositol (GPI)-anchored proteins, such as CD55 and CD59 [1]. Epidemiologic surveys report an incidence of 0.08-0.57 per 100,000 person-years and a prevalence of 38 per million [1]. Loss of these surface proteins leads to complement-mediated hemolysis, thrombosis, and marrow failure. Hemolysis in PNH is primarily driven by activation of the alternative complement pathway. Complement activation, exacerbated by stressors such as infection or surgery, can lead to breakthrough hemolysis (BTH) [2]. In advanced lung adenocarcinoma, particularly in cases harboring epidermal growth factor receptor (EGFR) gene mutations, EGFR tyrosine kinase inhibitors (EGFR-TKIs) are the standard first-line therapy. However, for patients who develop resistance or intolerance to EGFR-TKIs, platinum-based chemotherapy remains a key option. Combination therapy with carboplatin (chemotherapy regimen consisting of carboplatin, CBDCA) and pemetrexed (PEM) is an established standard regimen for advanced nonsquamous non-small cell lung cancer (NSCLC). In EGFR mutation-positive NSCLC resistant to EGFR-TKIs, the median progression-free survival (PFS) and overall survival with this regimen have been reported as 5.5 and 14.7 months, respectively [3]. This case report describes a patient with PNH who developed BTH induced by essential chemotherapy for life-threatening lung cancer. The introduction of ravulizumab, a complement C5 inhibitor, not only controlled hemolytic episodes but also coincided with an unexpectedly prolonged disease control of the lung cancer. This report describes a rare case and reviews the management of chemotherapy-induced BTH in PNH.

## Case presentation

**Figure 1:**
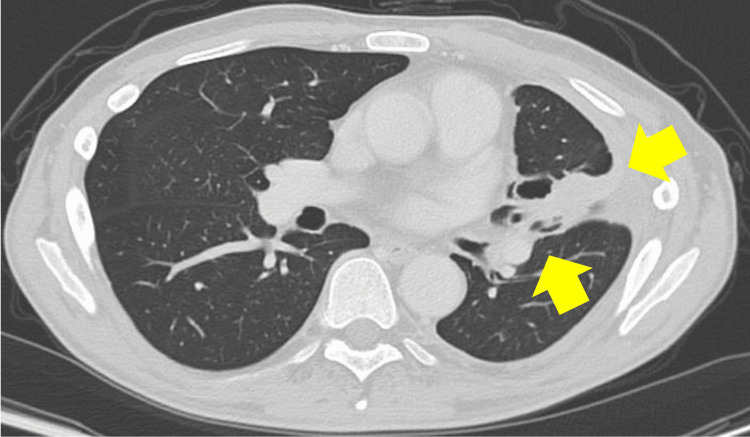
Chest CT at the time of ravulizumab initiation showing an irregular left upper lobe mass, carcinomatous pleurisy, and left hilar lymphadenopathy (yellow arrows) CT: computed tomography

A 52-year-old man with PNH diagnosed in his 30s had been observed without treatment. In May 2018, he developed exertional dyspnea, and a chest computed tomography (CT) scan in August revealed massive left-sided pleural effusion. Pathological examination of the pleural fluid led to a diagnosis of stage IVA EGFR-mutated lung adenocarcinoma. Osimertinib was initiated as first-line therapy in September 2018; however, frequent Grade 2-3 diarrhea made continuation difficult. In November 2020, following the discontinuation of osimertinib, a chest CT scan demonstrated enlargement of the primary lesion and new lymph node metastases. Consequently, combination chemotherapy with CBDCA and PEM was initiated as second-line treatment in December 2020. From February 2021, hemolytic parameters began to worsen, and by May 2021, laboratory tests showed a marked increase in lactate dehydrogenase (LDH) (peak: 2,462 U/L), a decrease in hemoglobin (Hb) (nadir: 4.6 g/dL), and elevated total bilirubin (peak: 2.7 mg/dL), leading to a diagnosis of BTH. The patient was hospitalized in May 2021 and started on ravulizumab. Key admission findings included marked anemia (Hb 4.6 g/dL), significantly elevated LDH (2,462 U/L), and low haptoglobin (3.8 mg/dL). Flow cytometry revealed a PNH red blood cell clone (CD55-/CD59-) comprising 14.9%. Total hemolytic complement activity (CH50) was elevated (≥60 U/mL), indicating robust complement activation. Chest CT demonstrated an irregular left upper lobe lesion, left-sided carcinomatous pleurisy, and left hilar lymphadenopathy. These laboratory and imaging findings at the time of ravulizumab initiation are presented in Table 1 and Figure 1.

**Table 1:** Key laboratory findings at the time of ravulizumab initiation WBC: white blood cell count; RBC: red blood cell count; Hb: hemoglobin; Plt: platelet count; TP: total protein; Alb: albumin; AST: aspartate aminotransferase; ALT: alanine aminotransferase; LDH: lactate dehydrogenase; T-Bil: total bilirubin; D-Bil: direct bilirubin; BUN: blood urea nitrogen; Cre: creatinine; Fe: serum iron; TIBC: total iron-binding capacity; C3 and C4: complement components 3 and 4; CH50: total hemolytic complement activity; CD55 and CD59: complement regulatory proteins expressed on erythrocytes

Investigations	Results	Normal range
WBC (/μL)	2,800	3,900-9,800
RBC (×10⁴/μL)	133	427-570
Hb (g/dL)	4.6	13.5-17.6
Plt (×10⁴/μL)	10.6	15.8-34.8
Reticulocyte (%)	1.7	0.2-2.7
TP (g/dL)	6.7	6.5-8.0
Alb (g/dL)	6.7	6.5-8.0
AST (U/L)	120	13-35
ALT (U/L)	20	13-35
LDH (U/L)	2,462	124-222
T-Bil (mg/dL)	2.7	0.3-1.2
D-Bil (mg/dL)	0.2	0-0.2
BUN (mg/dL)	13	8.0-22.0
Cre (mg/dL)	0.98	0.60-1.10
Haptoglobin (mg/dL)	3.8	19-170
Fe (μg/dL)	84	80-200
TIBC (μg/dL)	342	290-355
Ferritin (ng/mL)	1,106	13-277
C3 (mg/dL)	124.7	73-138
C4 (mg/dL)	26	11-31
CH50 (/mL)	>60	30-46
Erythrocytes surface markers		
CD55-/CD59- (%)	14.9	-
CD55-/CD59+ (%)	3	-
CD55+/CD59- (%)	0	-
CD55+/CD59+ (%)	82.1	-

**Figure 2:**
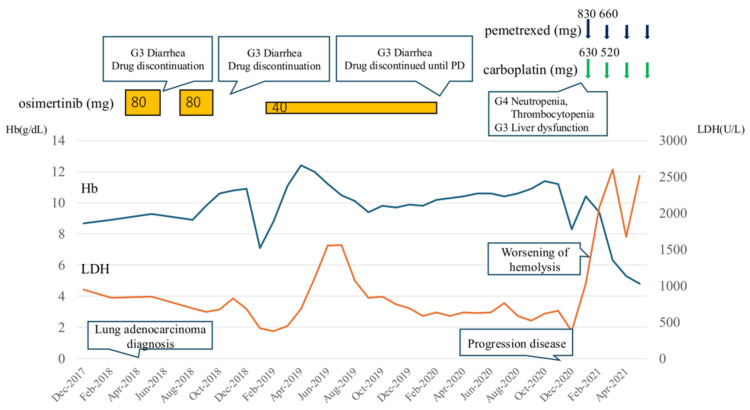
Clinical course of the patient, showing the period from lung cancer diagnosis, initial chemotherapy with osimertinib, and subsequent carboplatin plus pemetrexed leading to worsening hemolysis and introduction of ravulizumab Hb: hemoglobin; LDH: lactate dehydrogenase; PD: progression disease

**Figure 3:**
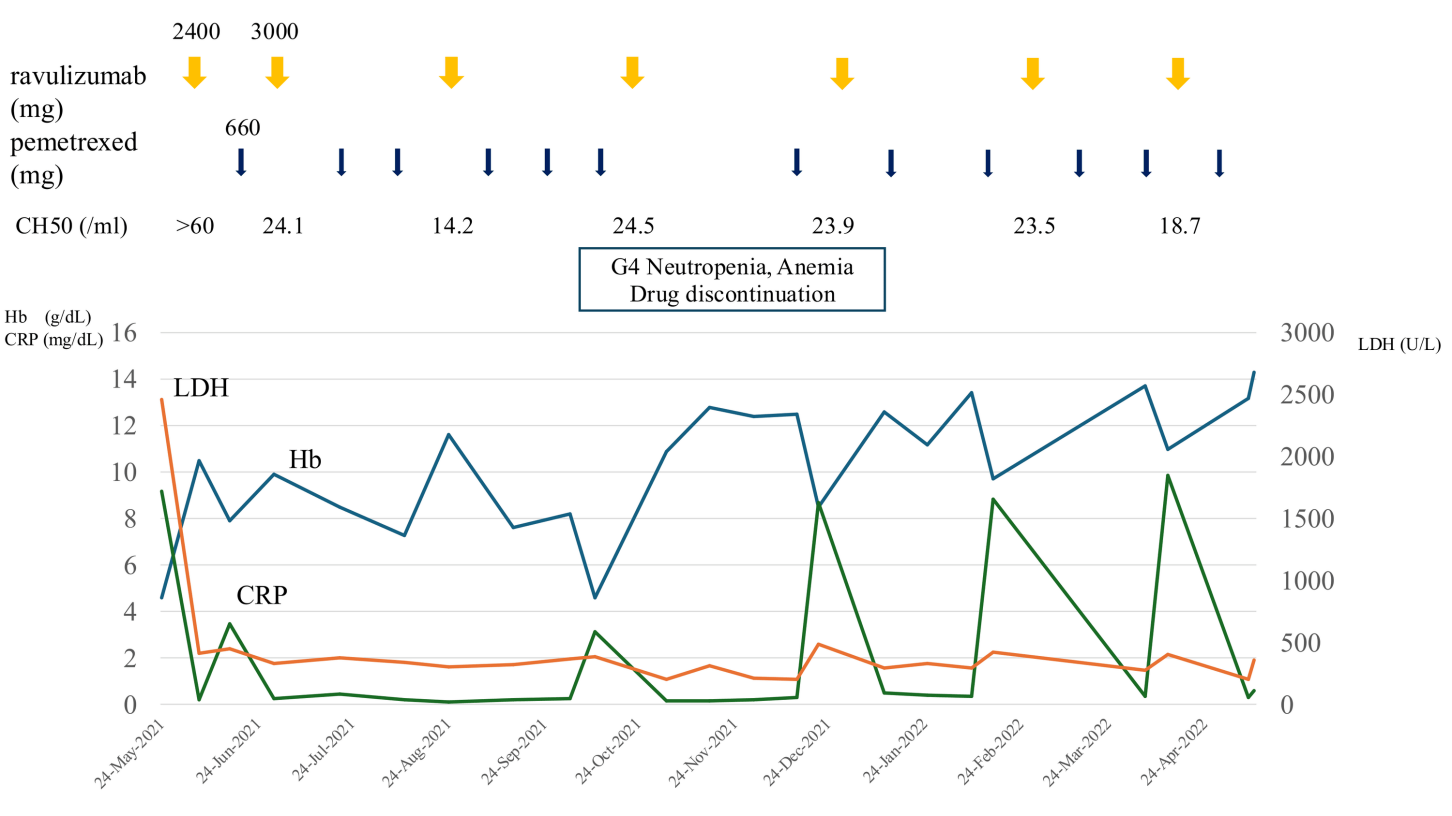
Clinical course of the patient, showing the initial phase after ravulizumab introduction, with continued pemetrexed administration (Pem→Rav sequence) and associated hematological changes Hb: hemoglobin; CRP: C-reactive protein; LDH: lactate dehydrogenase; CH50: total complement activity

**Figure 4:**
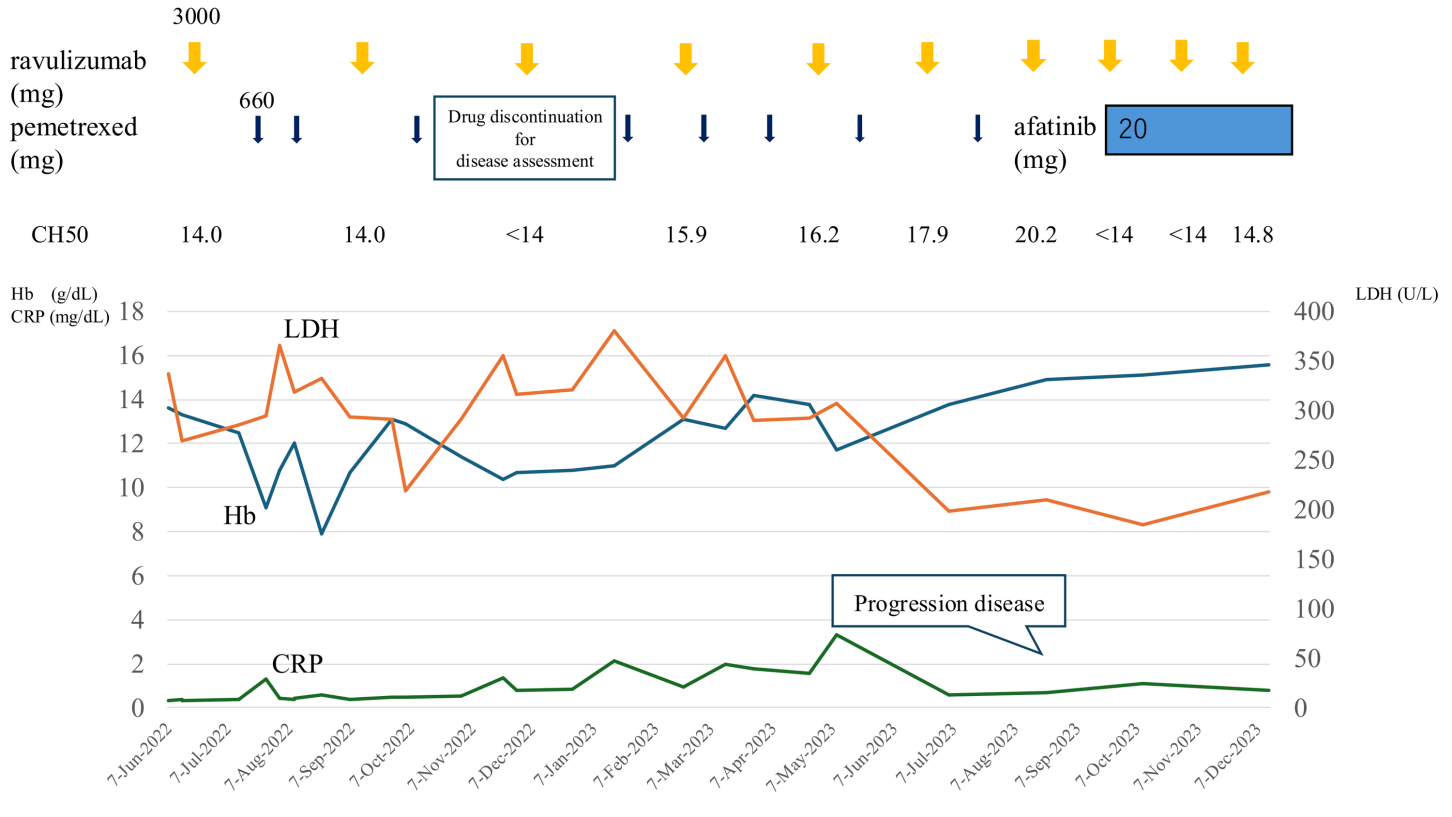
Clinical course of the patient, showing the later phase demonstrating the optimized sequence of ravulizumab prior to pemetrexed (Rav→Pem sequence), long-term disease control, eventual progression, and subsequent treatment with afatinib Hb: hemoglobin; LDH: lactate dehydrogenase; CRP: C-reactive protein; CH50: total complement activity

Following the initiation of ravulizumab, hemolytic findings improved rapidly. LDH decreased to approximately 250 U/L, and Hb recovered to 10.5 g/dL without transfusions. CH50 stayed below 14 U/mL. With hemolysis suppressed, PEM chemotherapy was safely resumed on an outpatient basis. However, mild hemolysis still occurred at the start of chemotherapy. Based on the observed pattern of hemolysis, the administration schedule was adjusted to give ravulizumab prior to each cycle of chemotherapy, which effectively prevented further hemolytic episodes. Two years and five months after starting CBDCA-PEM therapy, disease progression of the lung adenocarcinoma was confirmed by imaging studies. This duration substantially exceeded the median PFS of approximately 5.5 months for this regimen in previously treated advanced nonsquamous NSCLC [4]. In June 2023, afatinib was started. After this change, both Hb and LDH levels normalized, strongly suggesting that CBDCA, PEM, or both had triggered the BTH. The clinical course is summarized in Figures 2-4.

## Discussion

BTH precipitated by cytotoxic chemotherapy is rarely reported in PNH; we identified only three published cases (Table 2) [4-6].

**Table 2:** PNH manifestation or exacerbation after systemic anticancer therapy: a review of clinical characteristics in three cases PNH: paroxysmal nocturnal hemoglobinuria

Study	Cancer type	Cancer treatment	PNH status	PNH treatment/outcome	Cancer outcome
Lim et al. [4]	Cancer of unknown primary	Carboplatin-paclitaxel + erlotinib	Hemolytic crisis diagnosed with PNH	Eculizumab hemolysis markedly suppressed	No recurrence for >8 years (under eculizumab)
Sikuyayenga and Karlin [5]	Breast cancer	Aromatase inhibitor	Known PNH hemolytic crisis	Supportive care	Not specified
Hakim et al. [6]	Breast cancer	Bispecific antibody MDXH210	Hemoglobinuric acute renal failure suggestive of PNH	Not specified	Died from metastatic cancer

These reports suggest that cytotoxic chemotherapy, particularly regimens with pronounced myelosuppressive or proinflammatory effects, can precipitate BTH or reveal previously subclinical PNH. Inflammatory stress appears central to chemotherapy-induced BTH, as cytotoxic agents can promote the release of proinflammatory cytokines and induce systemic responses. Both CBDCA and PEM have been shown to upregulate proinflammatory cytokines and polarize macrophages toward an M1-like phenotype in vivo [7-9]. Such cytokine surges can amplify the activation of the alternative complement pathway, lowering the threshold for intravascular hemolysis in PNH. In our case, a sharp rise in C-reactive protein accompanied each episode of worsening hemolysis, supporting an inflammation-driven mechanism. Persistent complement activation was also reflected by a CH50 level of ≥60 U/mL on admission. These observations indicate that systemic inflammatory responses to cytotoxic chemotherapy can act as a potent trigger for BTH in susceptible individuals with PNH. Local acidosis represents an additional, noninflammatory mechanism that can precipitate BTH. The classical Ham test demonstrates that complement-mediated lysis of PNH erythrocytes is potentiated at pH 6.5-7. Carboplatin preparations are slightly acidic (pH 5-7), and rapid infusion can transiently reduce systemic or microvascular pH. Such acidification may facilitate assembly of the alternative pathway C3 convertase on GPI-deficient red cells, thereby accelerating intravascular hemolysis. We speculate that chemotherapy-related acidosis and the surrounding inflammatory environment acted synergistically in our patient to produce the unusually severe BTH episode. Prompt and durable C5 blockade proved essential to sustaining chemotherapy in this patient. Ravulizumab, a long-acting monoclonal antibody that blocks cleavage of complement component C5, quickly lowered LDH, raised Hb, and reduced CH50 to <14 U/mL. These laboratory changes show full terminal-complement inhibition and match the responses reported in phase 3 PNH trials [10,11]. After the initial response, mild hemolysis recurred at the start of each chemotherapy cycle. Administering ravulizumab prior to each cycle prevented further episodes, and the patient completed 14 cycles without transfusion or thrombosis. These findings underscore the value of tailored C5 inhibition during cytotoxic therapy in PNH. In this patient, lung adenocarcinoma remained progression-free for two years and five months with CBDCA-PEM plus ravulizumab, well beyond the approximately 5.5 month median PFS reported for this regimen in EGFR-TKI-resistant NSCLC [3]. A comparable case, an eight-year remission after eculizumab in cancer of unknown primary, has been described [4]; however, single-patient observations are insufficient to establish an antitumor benefit from C5 blockade. Complement fragments, especially C5a, reshape the tumor microenvironment in several reinforcing ways. Preclinical studies show that C5a recruits myeloid-derived suppressor cells, redirects macrophages toward an M2 phenotype, and increases vascular endothelial growth factor expression [12-17]. Separately, C5a signaling through C5aR1 on tumor cells can enhance cell survival, foster drug resistance, and facilitate immune evasion [18-20]. It remains unclear whether sustained C5 blockade mitigates these protumor pathways in solid tumors; targeted clinical trials are needed to address this point. Because this report describes only one patient, it remains uncertain whether C5 inhibition was responsible for the prolonged tumor control; other factors, such as the patient’s tumor biology or the CBDCA-PEM regimen itself, may have contributed. Even so, the case demonstrates that ravulizumab can suppress chemotherapy-induced severe BTH and keep treatment on track in PNH. Giving the drug before each cycle proved a practical way to avert additional hemolysis without interrupting therapy. The unusually long PFS observed here, together with the eight-year remission reported after eculizumab [4], indicates that it is worth investigating whether sustained complement blockade confers a direct antitumor benefit. Establishing optimal dosing schedules in cancer-complicated PNH and clarifying whether C5 inhibition adds value on its own or in combination with chemotherapy or immunotherapy represent important goals for future prospective studies.

## Conclusions

Ravulizumab rapidly controlled chemotherapy-induced BTH in a patient with PNH, enabling uninterrupted CBDCA-PEM treatment for lung adenocarcinoma. Schedule-adjusted dosing before each cycle prevented further episodes and supported a PFS of two years and five months, far longer than expected for this regimen. Although a single case cannot prove an antitumor effect of complement blockade, the experience highlights the practical value of C5 inhibition during intensive chemotherapy and justifies prospective studies in cancer-complicated PNH.
